# Significance of Exhaled Nitric Oxide and Carbon Monoxide in Auxiliary Diagnosis and Evaluation of Allergic Rhinitis

**DOI:** 10.1155/2022/2083057

**Published:** 2022-11-23

**Authors:** Chuanliang Zhao, Mali Qin, Ling Jin, Ju Lai, Yang Wang, Shuangxi Liu, Shaoqing Yu

**Affiliations:** ^1^Department of Otolaryngology Head and Neck Surgery, Tongji Hospital, School of Medicine, Tongji University, Shanghai 200065, China; ^2^Department of Allergy, Tongji Hospital, School of Medicine, Tongji University, Shanghai 200065, China

## Abstract

**Objective:**

The concentration of exhaled NO and CO is considered as a candidate marker of respiratory inflammatory disease. This report discusses the exhaled NO and CO in the auxiliary diagnosis and evaluation of allergic rhinitis (AR).

**Methods:**

60 AR patients from October 2017 to March 2019, compared with 30 healthy controls. The severity of AR disease was distinguished by symptom score. Both groups were tested for exhaled nitric oxide through the nose or mouth and exhale carbon monoxide through the mouth. AR patients received glucocorticoid nasal spray for 1 month and were tested again for nNO, eNO, eCO, and symptom score.

**Results:**

Before treatment, all the nNO, eNO, and eCO of the AR group were higher than the control group. There were differences in the severe and moderate subgroup: severe > moderate > mild. eCO was not significantly different between the mild and control groups. The nNO, eNO, and eCO levels were positively correlated with symptom score. After treatment, nNO decreased significantly in the three subgroups; eNO and eCO in the severe AR group decreased significantly. Drawing the ROC curve, the area under curve (AUC) of nNO is 0.978. The AUC of eNO and eCO was 0.786 and 0.577, respectively.

**Conclusion:**

The nNO, eNO, and eCO in the AR group are higher than healthy people, which positively correlated with the severity of AR symptoms. The detection of nNO, eNO, and eCO can monitor the changes of AR. The detection of nNO level as an indicator of AR auxiliary diagnosis has high accuracy.

## 1. Introduction

The increasing incidence of allergic rhinitis (AR) in recent years severely affects the patients' quality of life. In clinical work, we mainly diagnose AR by asking patients about their allergy history, clinical manifestations, and the results of invasive allergen testing. But the severity of AR mainly depends on the symptom score subjectively, and the accuracy of the evaluation results is poor due to the different tolerance of patients [1, 2]. Along with the concept presentation of “noninvasive detection technology,” more and more noninvasive and objective methods for evaluating the degree of airway inflammation were proposed. Breath detection is a widespread concern, owing to exhaled gas composition and its concentration that are different in various diseases or the same diseases with different severity, so the detection of some gas composition and its concentration in exhaled gas can diagnose and monitor the disease, providing a new idea for auxiliary diagnosis and disease assessment of AR [3, 4].

Nitric oxide (NO) is the first gas molecule found to be in the diagnosis of the disease. In 2005, the American Thoracic Society and European Respiratory Society (ATS/ERS) issued the exhaled oxide (eNO) measurement standard and reference range for people aged 4 to 17, and in 2011, eNO level test has been widely used in the diagnosis and efficacy evaluation of asthma [5–8]. Carbon monoxide (CO) is considered as a potential signal medium to play a role in vascular function and intracellular homeostasis at physiological concentration. Exhaled CO (eCO), similar to eNO, is considered as a candidate marker of respiratory inflammatory disease, reflecting pathophysiological conditions such as smoking status and inflammatory diseases of the lungs and other organs.

In order to further investigate the correlation between the levels of nNO, eNO, and eCO in nasal exhalation and the severity of AR, we measured the levels of nNO, eNO, and eCO in nasal exhalation. We also conducted data analysis to explore the role of the levels of nNO, eNO, and eCO in the auxiliary diagnosis and status monitoring of AR. Through the analysis of the relationship between the AR and the upper and lower airway exhaled endogenous CO and NO levels, we explore the auxiliary diagnosis and condition monitoring of AR by testing expired endogenous NO and CO.

## 2. Methods

### 2.1. Research Object

From October 2017 to March 2019, according to the Guidelines for Diagnosis and Treatment of Allergic Rhinitis (2015, Tianjin), patients who were diagnosed as allergic rhinitis in our outpatient department were selected. We set inclusion criteria and exclusion criteria and then set up the AR group and healthy control group. The AR group, according to the symptom score, was divided into three subgroups: mild, moderate, and severe. Both groups are nonsmoking. The study protocol was approved by the Ethics Committee of our hospital. All the research objects in this experiment participate voluntarily and sign the informed consent.

### 2.2. Inclusion Criteria

1. More than 2 (including 2) symptoms such as sneezing, water-like nasal discharge, nasal congestion and nasal itching, and last or accumulate for more than 0.5-1.0 hour every day, which can be accompanied by eye itching, conjunctival congestion, and other eye symptoms
2. Skin prick test and/or serum specific IgE (SIgE) positive
3. Symptoms ≥ 4 days/week
4. Inflammatory changes of nasal mucosa: pallor, edema, and nasal watery secretion
5. Objects can actively cooperate with relevant examination and treatment

### 2.3. Exclusion Criteria

1. Patients with asthma, nasal polyps, sinusitis, or other inflammatory diseases of the upper and lower airways
2. Patients with a history of nasal sinus surgery, markedly deviation of the septum leading to prolonged nasal congestion or significant abnormalities of the nasal anatomical structure
3. Patients with abnormal cardiopulmonary functions or long-term medication
4. Smokers, respiratory infections, and systemic or local use of glucocorticoid drugs within the last 4 weeks
5. Patients with allergic rhinitis were treated with systemic or local drugs such as antihistamine in the last 4 weeks

### 2.4. Symptom Scores

Symptom intensity is scored by the four-point method: 0 points: asymptomatic; 1-3 points: mild symptoms (mild and tolerable); 4-6 points: moderate symptoms (obvious but not enough to interfere with normal daily activities or sleep at night); and 7-10: severe symptoms (enough to interfere with daily activities or sleep at night). According to the result of the symptom score and disease severity, the AR group was divided into three subgroups: mild, moderate, and severe. The scores 0 and 3 were classified as the mild subgroup, 4 as the moderate subgroup, and 7 as the severe subgroup.

### 2.5. Determination of nNO

The Nakoulam respiratory analyzer SUNVOU-CA2122 (product No. SVP003170802024) was manufactured by Wuxi Shangwo Medical Electronics Co., Ltd. Strictly follow the instructions.

Empty the nitric oxide in the instrument, and then place the olive-shaped nose probe on one of the participants' front nostrils to connect the nasal catheter with the nose probe. Then, click the device to start, the instrument inhales gas continuously in the nasal cavity of the participant through the connecting tube (the speed is 10 ml/s), and then instructs the participant to continue whistling, at this time, the soft palate is closed, blocking the airway gas mixed with the nasal cavity until the end of the nasal cavity measurement display time. The unit is ppb.

### 2.6. Determination of eNO

Empty nitric oxide in instrument, let the participants sit, plug the filter and breathe into the filter until the inspection mark down to the lowest, then exhale, and guide the objects in the course of the breath as far as possible to keep the indicator in the designated areas. While the expiratory flow is50 ± 5 ml/s and expiratory pressure is 5-20 cm H_2_O, the soft palate is closed, blocking the nasal gas into the lower airway until the displayed time reaches the end. Unit is the ppb.

### 2.7. Determination of eCO

Empty carbon monoxide in instrument, let the participants sit, plug the filter, begin to breathe through the mouth, make the air without carbon monoxide into the lungs until totally, hold breath for 5 seconds, the pulmonary circulation takes away inhaled CO in the air, then begin to exhale, and guide the objects breath as far as possible to keep the indicator in the designated areas, while the soft palate is closed at this time, blocking the nasal gas into the lower airway until the displayed time reaches the end. Unit is the ppm.

### 2.8. Glucocorticoid Nasal Spray Method of Use in the AR Group

In patients with AR, we use glucocorticoid nasal spray, mometasone furoate nasal spray, for treatment without taking other drugs:

1. Clean the nasal cavity before using general glucocorticoid nasal spray, and shake the liquid well (exhale gently before spraying to clear the nostrils)
2. Tilt your head slightly forward, press the other nostril by hand (avoid spraying directly toward the nasal septum), and then squeeze the bottle with your thumb steadily and rapidly to spray the liquid in a fog into one side of the nasal cavity
3. Spray twice on each side of the nasal cavity every morning and evening for one month
4. At the end of glucocorticoid treatment, determine the nNO, eNO, eCO, and symptom scores again as described

### 2.9. Statistical Analysis

SPSS24.0 and Excel software are used for statistical processing of the data; the data were presented in the form of mean ± standard deviation (*x* ± *s*). The *t*-test is used to perform statistical tests on the general data and the level of test indicators; *p* < 0.05 was considered statistically significant; the Pearson correlation is used for correlation analysis, and *p* < 0.05 was considered statistically significant. The receiver operating characteristic (ROC) curve is used to evaluate the recognition ability of the nNO, eNO, and eCO for AR. The greater the area under curve (AUC) value is, the higher the accuracy of the auxiliary diagnosis of the corresponding indicators will be.

## 3. Results

### 3.1. Comparative Analysis of Basic Data

The AR group included 60 patients with allergic rhinitis, the control group included 30 healthy patients, baseline data (age, gender, height, and weight) of the AR group and control group are used for descriptive statistics using mean ± standard deviation (*x* ± *s*), and *p* values are all greater than 0.05 obtained by *t*-test, which means no statistically significant differences in the baseline data (Table 1) of the two groups.

### 3.2. Comparison of the nNO, eNO, and eCO Levels between the AR Group and Control Group

**Figure 1:**
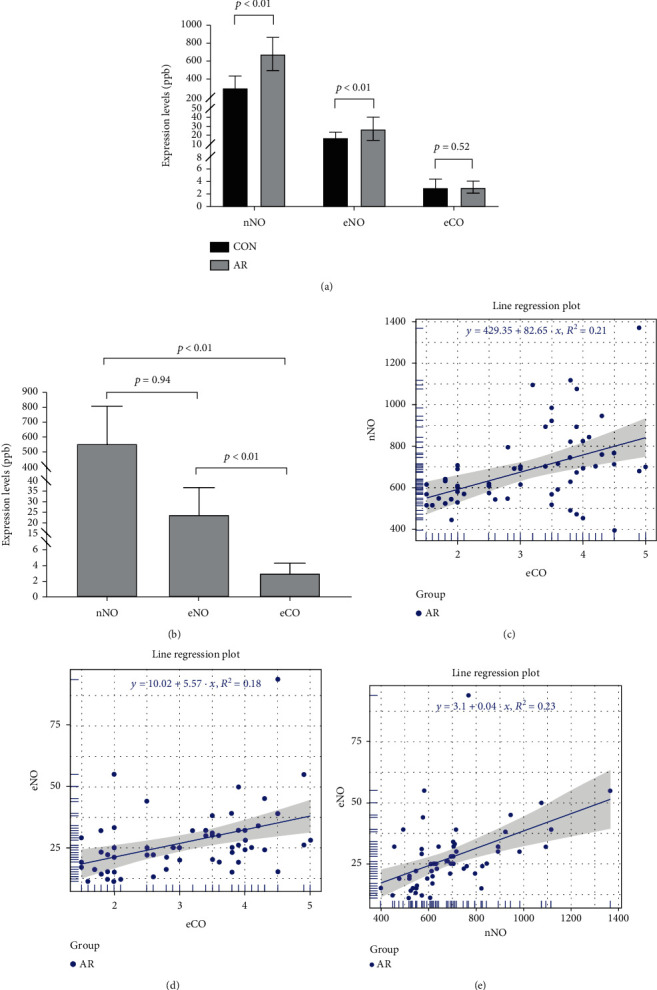
(a) Comparison of the nNO, eNO, and eCO levels between the AR group and control group (*x* ± *s*); (b) comparison of the nNO, eNO, and eCO expression levels (*x* ± *s*); (c) correlation analysis of nNO and eCO, *R*^2^ = 0.21, *p* < 0.01; (d) correlation analysis of eNO and eCO, *R*^2^ = 0.18, *p* < 0.01; (e) correlation analysis of eNO and nNO, *R*^2^ = 0.23, *p* < 0.01.

The results of nNO, eNO, and eCO show that the nNO level in the AR group is 685.83 ± 182.41 ppb, eNO level is 27.31 ± 13.40 ppb, and eCO level is 3.14 ± 1.04 ppb. The level of nNO, eNO, and eCO is increased compared with the control group, and the differences of nNO and eNO between the AR group and CON group are statistically significant (Figure 1(a)). In the analysis of the expression levels of nNO, eNO, and eCO, we found that the expression levels of nNO and eCO and eNO and eCO were significantly different, but there was no difference between the expression levels of nNO and eNO (Figure 1(b)). To study the correlation between the expression levels of nNO, eNO, and eCO, we performed the Pearson correlation analysis. And we found that the expression levels of nNO and eCO (*R*^2^ = 0.21, *p* < 0.01), eNO and eCO (*R*^2^ = 0.18, *p* < 0.01), and eNO and nNO (*R*^2^ = 0.23, *p* < 0.01) have a certain correlation (Figures 1(c) and 1(e)). Although *R*^2^ was less than 0.5, *p* was less than 0.01.

### 3.3. The Levels of nNO, eNO, and eCO in the AR Group Are Correlated with the Severity of the Symptoms

**Figure 2:**
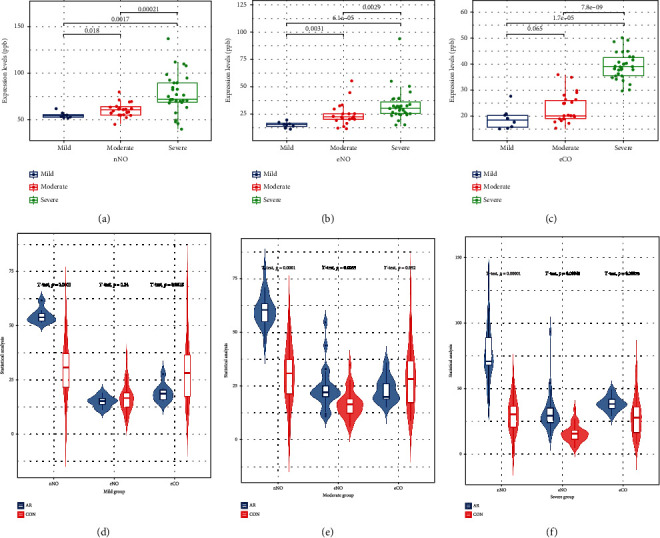
The levels of nNO, eNO, and eCO in the three subgroups of AR were higher than those in the control group, and the differences were statistically significant except for eNO in mild AR group (*p* = 0.24) and eCO in moderate AR group (*p* = 0.052).

According to the AR condition rating scale, the AR group was divided into three subgroups: severe, moderate, and mild, according to the results of the symptom score and the severity of the disease. We found that the levels of nNO, eNO, and eCO in severe AR subgroups were higher than those in moderate and mild AR subgroups. However, there was no difference between the control group and mild AR subgroup in eNO and eCO, and there was no difference in eCO in moderate AR subgroup, with *p* value greater than 0.05 (Figure 2).

### 3.4. Correlation Study of the nNO, eNO, and eCO with Symptom Scores in AR Group

**Figure 3:**
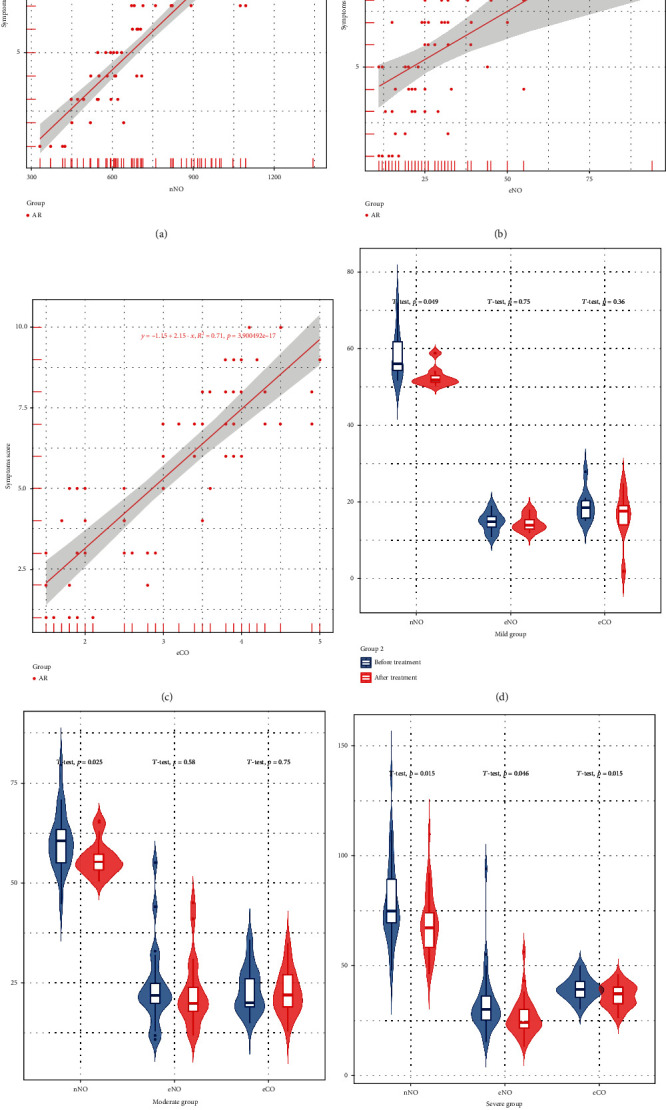
Correlation between nNO, eNO, eCO, and symptom score in AR group. (a) The correlation between nNO level and symptom score, *R*^2^ = 0.8, *p* = 0.01; (b, c) although there was a positive correlation between eNO, eCO, and symptom score, the *R*^2^ of the two groups was 0.2 and 0.71. (d) The nNO of allergic rhinitis patients in the three groups was significantly lower than that before treatment, with statistical significance; (e, f) although eNO and eCO decreased after treatment, only in severe patients, the changes were statistically significant.

To study the correlation between the severity of AR symptoms and the level of nNO, eNO, and eCO, we used the Pearson correlation analysis to show that the nNO level was positively correlated with the symptom score in AR patients (*R*^2^ = 0.8, *p* < 0.05), the eNO level was positively correlated with the symptom score in AR group (*R*^2^ = 0.2, *p* < 0.05), and the eCO level was positively correlated with the symptom score in AR group (*R*^2^ = 0.7, *p* < 0.05) (Figures 3(a)–3(c)).

### 3.5. Changes of nNO, eNO, and eCO after 1 Month of Glucocorticoid Nasal Spray Treatment

We measured the levels of the nNO, eNO, and eCO in the mild, moderate, and severe subgroups before and after treatment and analyzed the significance by paired *t*-test. The levels of the nNO, eNO, and eCO were significantly decreased in patients with severe AR after treatment compared with those before treatment with glucocorticoid. In the moderate AR group, only nNO level was decreased, while eNO and eCO levels were not statistically significant. The changes of nNO, eNO, and eCO level in patients with mild AR were not statistically significant (Figures 3(d)–3(f)).

### 3.6. The nNO, eNO, and eCO Were Used as Indicators for Auxiliary Diagnosis of AR, and Draw the ROC Curve

**Figure 4:**
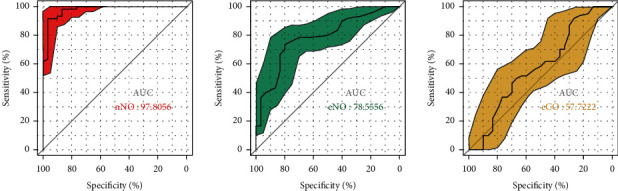
ROC curve of the nNO, eNO, and eCO levels for AR diagnosis. The AUC of the three indicators is all higher than 50%, which proved that the three indicators are both valuable in the detection of AR. The AUC of nNO is 97.8%, which is close to 100%. It shows that nNO has high value in AR detection.

The ROC curve was drawn using nNO value as an indicator for auxiliary diagnosis of AR (the area under the curveAUC = 0.978,*p* < 0.001). When the boundary value of the nNO measurement has taken 516 ppb, the sensitivity and specificity are both high (91.7%, 96.7%), while the specificity was 96.7%. The ROC curve was plotted alike using eNO and eCO values as diagnostic indicators of AR (the area under the curveAUC = 0.786,*p* < 0.001, and (AUC = 0.577,*p* = 0.445) (Figure 4). As an indicator for auxiliary diagnosis, the accuracy was relatively low.

## 4. Discussion

AR, as the most common type of rhinitis, is a type I allergic disease mediated by IgE. The clinical diagnosis of AR is mainly based on the patient's typical history of allergy, present symptoms, and invasive allergen test results [9, 10], but this diagnostic method of classic has some defects, such as invasive testing that is not well accepted by patients, and the determination results unable to distinguish the severity of the disease still need to be graded according to the symptom score. But this symptom score for severity is subjective and ignores the assessment of inflammation in the upper and lower airways. However, the long-term existence of upper and lower airway inflammation will cause irreversible changes in the upper and lower airway microenvironment, leading to the onset of chronic sinusitis, asthma, etc. [2, 9]. In view of this, it is of great significance for clinical diagnosis and treatment to find a practical and objective method to monitor the respiratory inflammation of AR patients.

Noninvasive airway inflammation has gained increasing attention with the introduction of the concept of “inflammometry.” More and more researchers are involved in the field. In addition, NO is synthesized from L-arginine catalyzed by nitric oxide synthase with high lipid solubility and active properties. Endogenous NO can play a physiological role in vascular tension and homeostasis, such as NO produced in the upper and lower airways that can inhibit the growth of bacteria, viruses, fungi, and tumor cells; therefore, it can protect the upper and lower airways [11]. Endogenous CO is also a kind of low molecular weight gas. Although the discovery of endogenous CO is more than 20 years earlier than that of endogenous NO, it had been regarded as a waste product generated in the body for a long time. As a result, CO was ignored by researchers. Studies in recent decades have attempted to determine the physiological significance of endogenous CO production, which includes the gas's role in vascular function, inflammation, and neuronal signaling mediator. The study by Shaoqing et al. reported that HO-1 level in AR guinea pigs was positively correlated with endogenous CO and the endogenous CO level of AR guinea pigs was higher than that of the control group [12]. In recent years, it has been found that the levels of NO and CO in the exhaled breath of AR patients were increased. The detection of endogenous level of NO and CO in exhale for AR diagnosis and condition monitoring has attracted more and more attention from researchers in the field of otolaryngology.

The results of this study showed that the nNO level of AR group was higher than that of the control group, and the difference was statistically significant. At the same time, the nNO level of patients with mild AR was also higher than that of the control group, which was consistent with the conclusions of other studies. The increase of nNO level in AR has a variety of pathophysiological effects, including vasodilation and changes of sensory nerve endings, so it aggravated nasal congestion, nasal leakage, and sneezing. In other words, the higher the nNO level, the more severe the symptoms would be. As shown in the results of this study, the nNO level of the severe AR subgroup was higher than that of the moderate and mild AR subgroups (*p* < 0.05), so did the correlation analysis showing that the nNO level was positively correlated with the severity of the symptoms of AR patients (*r* = 0.506, *p* < 0.05). But Lee et al. and Baraldi et al. study the nNO level by comparing different severity of patients with AR and found that the nNO level of patients with severe AR is below than that of moderate AR patients, while the conclusion was that there was no significant correlation between the level of nNO and the severity of the symptoms in AR patients [13, 14]. This contradiction that we consider may be that severe AR patients had serious nasal and sinus mucosa edema, which caused the sinus and nasal congestion; thus, NO diffusion is blocked and lowers the detection of nNO. And this study also detected that the nNO in sectional patients with severe AR was not high, even lower than the average level of the normal control group, but these kinds of patients with severe AR were less in our study. In view of this, we recommend that clinicians use nNO as monitoring control in AR patients, necessarily nNO level should be combined with symptom severity. We also compared and analyzed the differences between the expression levels of nNO, eNO, and eCO and found that nNO/eCO and eNO/eCO have significant statistical differences, but the expression levels of nNO/eNO had no statistical difference. The exhaled CO is similar to the exhaled NO, which is considered to be a candidate marker of respiratory inflammatory disease. So the differential expression levels of nNO/eCO and eNO/eCO may play an important role in the diagnosis of AR. The level of nNO/eNO may have homology, so there is no statistical difference.

The eNO level in AR group was higher than those in the normal control group (*p* < 0.05), and eNO level in severe AR subgroup was higher than those in patients with moderate or mild AR (*p* < 0.05), and eNO level was positively correlated with the severity of the symptoms (*R* = 0.461, *p* < 0.05) [15, 16]. Lee et al. study the eNO by comparing patients with allergic rhinitis (without asthma) and normal control group and found that the eNO level of patients with AR was higher than that of the control group, while the eNO level of patients with severe AR is higher than that of patients with mild and moderate AR [13]. Baraldi et al. study the eNO level by comparing the seasonal allergic rhinitis patients and healthy volunteers, and the eNO level in symptomatic and asymptomatic AR patients was found to be higher than those in the control group; moreover, the eNO level in symptomatic AR patients was higher than those in asymptomatic AR patients [14].

There was no significant difference in the level of eCO between the mild and moderate AR patients and the control group, which was consistent with the study of Shaoqing et al. [12]. It may also be that the inclusion criteria in the experiment are not strict enough or the operation of eCO level detection is not strict enough, because the determination of eCO level is easily affected by many factors, including the weight, age, gender, and lower airway inflammatory diseases of the subjects [15]. Through this study, we can see that in the role of auxiliary diagnosis and disease monitoring of AR, the role of the determination of eCO level in AR disease monitoring needs to be further studied, and its value in clinical application needs to be further improved.

In this study, the detection of nNO level is used as an indicator for AR auxiliary diagnosis, and ROC curve is drawn. The area under the curve reached 0.978. When the threshold value is 516 ppm, the sensitivity and specificity were both high, 91.7% and 96.7%, respectively. Using the detection of eNO and eCO as auxiliary diagnostic indicators of AR, ROC curve is drawn, the area under the curve was 0.786 (*p* < 0.001) and 0.577 (*p* = 0.445), respectively. Compared with the nNO, the accuracy of eNO and eCO for AR diagnosis was relatively low. In the study of Lee et al., the nNO and eNO levels were detected to assist the diagnosis of AR, and ROC curve was drawn with AUC of 0.8527 and 0.6463, respectively [13], consistent with the conclusion of this study. In view of this, after excluding asthma, nasal polyps, and sinusitis, which were the upper and lower airway inflammatory diseases, other serious nasal septum deviation or other interference factors, compared with the eNO and eCO, detect the nNO as an index of auxiliary AR diagnosis having higher accuracy, and because of its simple operation, strong repeatability, noninvasive, and no side effect, it is worth popularizing widely in clinical.

We believe that AR patients may have chronic lower airway inflammation without clinical symptoms, and the higher the degree of inflammation in AR patients and the higher the eNO in AR patients, it is speculated that the greater the possibility of AR patients complicated with chronic lower airway inflammatory disease. Takeno et al. and Olin et al. research the correlation between the eNO level and occurrence of wheezing in the general population reported. The elevated NO level in the lower and middle airways of objects with no respiratory symptoms as a sign of subclinical airway inflammation, heralding the small airway inflammation exists, will be a risk factor for the development of asthma [17, 18]. In patients with allergic rhinitis without asthma, the respiratory physician used eNO as a diagnostic tool and efficacy monitoring indicator for asthma, when the eNO increased, suggesting that the patient should be diagnosed and treated with AR at the same time.

## Figures and Tables

**Table 1:** Comparison of patient information between the AR group and control group (*x* ± *s*).

Patient information	AR group	Control group	*p*
Age	25.0 ± 6.3	27.0 ± 8.4	*p* = 0.365
Gender	24/36	13/17	*p* = 0.705
Height	174.3 ± 9.2	176.2 ± 7.5	*p* = 0.665
Weight	68.2 ± 11.0	67.1 ± 8.8	*p* = 0.831

## Data Availability

The datasets used during the current study are available from the corresponding authors on reasonable request.
